# ENGAGING AFRICAN AMERICAN FAMILIES IN END OF LIFE DISCUSSIONS: CHALLENGES AND FACILITATORS

**DOI:** 10.1093/geroni/igz038.3449

**Published:** 2019-11-08

**Authors:** Pamela Z Cacchione, Le'Roi L Gill, Justine Sefcik

**Affiliations:** 1 University of Pennsylvania School of Nursing, Senior Fellow , Leonard Davis Institute of Health Care Economics, Philadelphia, Pennsylvania, United States; 2 Mercy LIFE West Philadelphia, Philadelphia, Pennsylvania, United States; 3 University of Pennsylvania, Philadelphia, Pennsylvania, United States

## Abstract

Our previous study, African American Preferences Around End of Life, identified that AA Elders wanted to talk to their family about their preferences, but their family tended to avoid discussing end of life topics. We found that African American families often have a difficult time broaching the subject of end of life for a variety of emotional, cultural and religious reasons. Therefore, the purpose of this qualitative descriptive study was: To better understand the challenges and facilitators that influenced end of life conversations within the African American family. Methods: In this qualitative descriptive study, we interviewed 15 AA family caregivers of older adults. Participants were family members of older adults enrolled in an urban Program of All-inclusive Care for the Elderly. Individual interviews lasted on average 50 minutes. Data analysis was completed using conventional content analysis. Results: The majority of participants were between 55 - 65 years of age and adult children of the AA older adult. Two themes emerged for challenges: I’m not comfortable and We just don’t talk about it. For facilitators again, two themes emerged: Another person took the initiative (e.g. health care provider led the conversation) and participants’ previous experience with death led them to initiate EOL conversations. In addition, three participants reported that after participating in the interview they planned to talk to their loved one to find out their end of life preferences. The results of this study provide insight into how health care providers can facilitate these important end of life preferences conversations.

